# The Crotoxin:SBA-15 Complex Down-Regulates the Incidence and Intensity of Experimental Autoimmune Encephalomyelitis Through Peripheral and Central Actions

**DOI:** 10.3389/fimmu.2020.591563

**Published:** 2020-10-28

**Authors:** Morena Brazil Sant'Anna, Aline C. Giardini, Marcio A. C. Ribeiro, Flavia S. R. Lopes, Nathalia B. Teixeira, Louise F. Kimura, Michelle C. Bufalo, Orlando G. Ribeiro, Andrea Borrego, Wafa H. K. Cabrera, Julio C. B. Ferreira, Vanessa O. Zambelli, Osvaldo A. Sant'Anna, Gisele Picolo

**Affiliations:** ^1^ Laboratory of Pain and Signaling, Butantan Institute, Sao Paulo, Brazil; ^2^ Institute of Biomedical Sciences, University of Sao Paulo, Sao Paulo, Brazil; ^3^ Laboratory of Immunogenetics, Butantan Institute, Sao Paulo, Brazil; ^4^ Department of Chemical and Systems Biology, School of Medicine, Stanford University School of Medicine, Stanford, CA, United States; ^5^ Department of Anesthesiology, Perioperative, and Pain Medicine, Stanford University School of Medicine, Stanford, CA, United States; ^6^ Laboratory of Immunochemistry, Butantan Institute, Sao Paulo, Brazil

**Keywords:** crotoxin, SBA-15, mesoporous silica, experimental autoimmune encephalomyelitis (EAE), IL-17, neuroinflammation, motor impairment

## Abstract

Crotoxin (CTX), the main neurotoxin from *Crotalus durissus terrificus* snake venom, has anti-inflammatory, immunomodulatory and antinociceptive activities. However, the CTX-induced toxicity may compromise its use. Under this scenario, the use of nanoparticle such as nanostructured mesoporous silica (SBA-15) as a carrier might become a feasible approach to improve CTX safety. Here, we determined the benefits of SBA-15 on CTX-related neuroinflammatory and immunomodulatory properties during experimental autoimmune encephalomyelitis (EAE), an animal model of multiple sclerosis that replicates several histopathological and immunological features observed in humans. We showed that a single administration of CTX:SBA-15 (54 μg/kg) was more effective in reducing pain and ameliorated the clinical score (motor impairment) in EAE animals compared to the CTX-treated EAE group; therefore, improving the disease outcome. Of interest, CTX:SBA-15, but not unconjugated CTX, prevented EAE-induced atrophy and loss of muscle function. Further supporting an immune mechanism, CTX:SBA-15 treatment reduced both recruitment and proliferation of peripheral Th17 cells as well as diminished IL-17 expression and glial cells activation in the spinal cord in EAE animals when compared with CTX-treated EAE group. Finally, CTX:SBA-15, but not unconjugated CTX, prevented the EAE-induced cell infiltration in the CNS. These results provide evidence that SBA-15 maximizes the immunomodulatory and anti-inflammatory effects of CTX in an EAE model; therefore, suggesting that SBA-15 has the potential to improve CTX effectiveness in the treatment of MS.

## Introduction

Multiple sclerosis (MS) is a quite frequent neurological disorder diagnosed in young adults. The common symptoms include, among others, fatigue, visual impairment, movement and coordination problems, cognitive dysfunction and pain (1). This chronic inflammatory disease is mediated mainly by auto-reactive T-lymphocytes, being classified as an autoimmune condition. The hallmarks of MS are myelin and neuroaxonal lesions, resulting from the increase in the blood-brain barrier (BBB) permeability by pro-inflammatory cytokines and chemokines action, which allows the influx of immune cells into the central nervous system (CNS) (2, 3). In addition to the increased infiltrating cells, activation and proliferation of resident glial cells are also observed (4–6). It is well known that T helper (Th) 17 plays a key role in the MS development, being involved in the BBB breach, glial cell activation and pro-inflammatory cytokine release, such as interleukin (IL) 17. The importance of Th17 cells in the development of the disease was also demonstrated in the experimental autoimmune encephalomyelitis (EAE) model, an animal model of MS (7–9).

Studies have shown that the EAE model reproduces neuroinflammation, axonal demyelination and motor impairment observed in MS, encouraging its use in studies aiming to understand the mechanisms involved in the course of the disease as well as to evaluate possible therapies (10, 11). Additionally, within this model, the first symptom to emerge is pain (hypernociception) followed by motor impairment (clinical signs) (12–14), which allows for the independent evaluation of both symptoms (15, 16). Treating these symptoms remains a challenge; current therapies are focused on modulating disease progression and evidence of a definitive cure is lacking (17, 18).

Natural products have been considered a rich source of compounds for the development of potential drugs for the treatment of pain and other disorders, such as neurodegenerative diseases (19, 20). Crotoxin (CTX), a neurotoxin from the *Crotalus durissus terrificus* rattlesnake, is the main compound responsible for the venom high toxicity. Despite the toxic effect, in low doses, it has prolonged anti-inflammatory, immunomodulatory, antitumoral and antinociceptive activities, making it a potential drug for chronic pain and inflammatory diseases (21–25). However, its toxic effect may comprise its usefulness in treating debilitating diseases or its ability to be administered in high doses.

Reports have demonstrated the great potential of mesoporous materials in the field of nanoscience originating from their particular characteristics such as unique porous structures and adsorption properties (26–30). Among these materials, mesoporous silica SBA-15 has been studied as a carrier agent, due to its physicochemical and structural properties, allowing it to act as a delivery system, aiding the release profile, and improving the immune response (31–34). Additionally, studies show that the SBA-15 surface allows for high drug-loading, which may reduce the toxicity of compounds (35, 36). Although the mechanisms by which SBA-15 decreases the toxicity of compounds is not clear, the controlled release of them may contribute to that. In accordance, previous studies from our group have demonstrated that SBA-15 allows an increase of CTX dose with non-toxic effects (37).

Considering that, in the present study, we proposed to improve the effect of CTX on the EAE model through its conjugation with silica SBA-15 and to investigate its effect on central and peripheral important sites for disease development and maintenance. Of interest, skeletal muscle contractility properties were also investigated, since peripheral alterations induced by EAE are poorly reported. Hence, the effect of the treatment with CTX conjugated to SBA-15 in the EAE model was evaluated and compared to unconjugated CTX using different schedules of treatment [single or 5 administrations, being 1 dose *per* day for 5 consecutive days] through behavioral and functional assays and molecular biology tools.

## Materials and Methods

### Animals

Experiments were performed on female C57BL/6 mice (38) (wild type, 18-22g), aged 8-12 weeks, from the animal facility of Butantan Institute (Sao Paulo, Brazil). Mice were group-housed in 5 to 6 animals per cage and kept under a 12-h light/dark cycle (lights on at 6 am) with food and water *ad libitum*. The animal room was kept under 21 ± 2.0°C and humidity (50% ± 10% RH). All behavioral tests were performed between 7:00 am and 5:00 pm. The experimental procedures performed on animals were approved by the Institutional Animal Care Committee of the Butantan Institute (CEUAIB, protocol number 1320/14 and 7986010819), and performed in accordance with the National Council of Animal Experimentation Control (CONCEA) regulation and with the guidelines for the ethical use of conscious animals in pain research published by the International Association for the Study of Pain (39). Behavioral tests were performed with 8 animals per group; histological and functional skeletal muscle assays included 4-5 animals per group; interleukins expression or release and TCD4^+^ cell frequency were analyzed using 5-6 animals per group.

### Induction of EAE and Clinical Signal Scale

Mice were randomly immunized subcutaneously (s.c.) with incomplete Freund’s adjuvant (#*F5506*, *IFA; SIGMA, St Louis, USA*) containing 4 mg/ml of *Mycobacterium tuberculosis* (#BD-231141, *H37Ra; Difco™*) and 200 μg of MOG_35–55_ (MEVGWYRSPFSRVVHLYRNGK peptide, Proteimax, Brazil) for the induction of EAE. Immediately after immunization and 48 h later, the animals received intraperitoneal (i.p.) injection of 300 ng of pertussis toxin (*#*P7208*, Sigma-Aldrich*). Control groups consisted of animals injected with CFA (incomplete Freund’s adjuvant containing 4 mg/ml of *Mycobacterium tuberculosis*) and pertussis toxin or naïve. Clinical scores were recorded daily by a blind experimenter and when a score of 3 or higher was observed, water and food were made available to the base of the cage to be easily accessed. Clinical scores were classified as follows in **Table 1** and reached the peak between the 14^th^ and 18^th^ day after immunization.

**Table 1:** Clinical EAE scores.

Score	Observation
0	No symptom
0.5	Partial loss of the tail tone
1.0	Complete loss of tail tone, with no evidence of hind limb weakness
1.5	Evidence of hip weakness upon ambulation (slightly wobbly)
2.0	Hip weakness and hind limb paresis
2.5	Partial hind limb paralysis (from one of the limbs)
3.0	Complete hind limbs paralysis, but capable of moving around the cage
3.5	Complete hind limbs paralysis and difficult to moving around the cage (forelimbs paresis)
4.0	Complete hind limbs paralysis and partial paralysis of the forelimbs, but still responsive (consider euthanasia)
4.5	Complete hind limbs paralysis and paralysis of the forelimbs, decreased responsiveness (consider euthanasia)
5.0	Immobile and unresponsive, moribund or death (immediate euthanasia)

### Crotoxin (CTX)

Crotoxin was purified from *Crotalus durissus terrificus* venom obtained from Laboratory of Herpetology, Butantan Institute, by anion-exchange chromatography as previously described (40, 41). Dr. Sandra Coccuzzo Sampaio from the Laboratory of Pathophysiology, Butantan Institute, kindly supplied CTX.

### SBA-15 Synthesis

The synthesis recipe and adsorption characterization of the SBA-15 were analogous to those reported elsewhere (42). Small-angle X-ray scattering [SAXS] characterization was performed as previously described (43).

### Preparation of the Complex (CTX:SBA-15) and Treatment

The CTX was diluted in phosphate-buffered saline (PBS) pH 7.4, slowly added to the silica SBA-15 (1:10) and held for 24 h at 2°C to 8°C, with occasional stirring.

In the *in vivo* assays, the following compounds were used: 40 μg/kg of CTX (22, 44) or 54 μg/kg of CTX:SBA-15 administered by subcutaneous (s.c., 200 μl) route, since SBA-15 conjugation enabled an increase of 35% of CTX non-toxic dosage, as previously demonstrated by our group (37). The animals were treated with 5 doses (1 administration/day for 5 consecutive days) or with a single administration, starting from the 5^th^ day post-immunization, based on previous results from our group which considered the onset of the first symptom of the disease to start the treatment (pain threshold alteration that occurred in the 4^th^ day after immunization) (45). PBS (s.c., 200 μl) was used as vehicle control.

### Behavioral Test for Mechanical Hypernociception Determination—Electronic von Frey

In a quiet room, animals were placed individually inside acrylic cages on an elevated wire grid and the plantar surface of the paw was stimulated with a pressure-meter which consisted of a hand-held force transducer fitted with a 0.5 mm^2^ polypropylene tip (*electronic von Frey anesthesiometer, Insight Equipment’s Ltda., Ribeirão Preto, SP, BRA*). Mice were first habituated to the experimental environment (room and apparatus) for at least 20 min. The mechanical nociception test consisted of applying a rising perpendicular force to the hind paw followed by a clear flinch response after a paw withdrawal. Paw stimulation is repeated until the animal shows three similar measures (46). The animals were submitted to pain sensitivity analysis before (baseline measurement) and every day after immunization with MOG_35-55_ by a blind experimenter until the first clinical signs of motor alterations appeared.

### *Ex Vivo* Skeletal Muscle Contractility Properties

The contractile properties (tetanic force) of the extensor digitorum longus muscle (EDL) were evaluated as previously described (47). Briefly, animals were euthanatized at the peak of EAE, the entire hindlimb was collected and the EDL muscle was isolated and placed in an organ bath containing 20 ml of Tyrode solution (2 mM CaCl_2_, 5 mM KCl, 1 mM KH_2_PO_4_, 1 mM MgSO_4_, 137 mM NaC_l_, 24 mM NaHCO_3_, 11 mM glucose, pH 7.4) at 25 °C perfused with carbogen (95% O_2_ + 5% CO_2_). The muscle was tied by the tendons to an apparatus attached to a force transducer (Grass Instruments model FT03, USA). The muscle optimal length was determined and the EDL was stimulated to contract isometrically through electrical field stimulation (Grass Instruments S-88 Grass-stimulator). The force transducer was recorded and analyzed using a PowerLab system (AD Instruments, USA). Forces are expressed in grams and normalized by the EDL weight.

### Skeletal Muscle Cross-Sectional Area (CSA)

The skeletal muscle cross-sectional area was assessed as previously described (48). Briefly, after euthanasia at the peak of EAE, the tibialis anterior muscle was collected, frozen, and stored in liquid nitrogen. Following that the muscle was fixed and cross-sectioned (10 μm sections) using a cryostat. Sections were submitted to HE-staining according to the procedure previously described (49). The myofiber cross-sectional area (μm2) was evaluated at 200 magnification and analyzed (Image Pro-Plus, NHI, USA). An investigator who was blind to the animal identity performed all of the analyses.

### Interleukins mRNA Analysis by Real-Time PCR

After euthanasia, the lymph nodes (pool of inguinal and mesenteric) and tissue from the region that included the lumbar segments of the spinal cord were collected. A time course of IL-17 mRNA expression induced by EAE was evaluated in both lymph nodes and spinal cord (3^rd^, 7^th^, 10^th^, and 14^th^ day). The timepoint of mRNA expression increasing was determined in each region, and it was selected for cytokine release evaluation and TCD4^+^ cell frequency analysis. The total RNA was isolated using the Illustra RNAspin Mini Kit (*GE Healthcare*), and the preparation was suspended in 20 μL of H_2_O (RNA free). RNA concentrations were determined using the NanoVue® apparatus (GE Healthcare Life Science) and 500 ng was transcribed into cDNA by SuperScript™ III reverse transcriptase *(#18080044, Thermo Fisher Scientific*), according to the manufacturer’s protocols. Quantitative real-time PCR was performed using gene-specific primers. Real-time PCR amplification mixtures contained 2 μl cDNA, 5 μl Fest SYBR™ Green Master Mix *(#4385612, Thermo Fisher Scientific*), 2.2 μl H_2_O (RNA free), and 0.8 μM specific PCR primers. Reactions were carried out in a StepOnePlus™ Real-Time PCR system (*Thermo Fisher*) thermocycler. The results were analyzed using the method of quantitative relative expression 2^−ΔΔCt^ (50). The primer sequences were as follows in **Table 2**.

**Table 2:** Cytokine levels evaluation by MULTIPLEX.

Gene	Forward sequence	Reverse sequence
Ppia	AGCGTTTTGGGTCCAGGAAT	AAATGCCCGCAAGTCAAAAG
HPRT	CGTCGTGATTAGCGATGATGA	CCAAATCCTCGGCATAATGATT
IL-17	GCGTGTCCAAACACTGAGGCCA	ATTGCGGTGGAGAGTCCAGGGT
IL-10	CCCAAGTAACCCTTAAAGTCCTG	GCTGGACAACATACTGCTAACC

### Cytokine Levels Evaluation by MULTIPLEX

The spinal cord from the region comprised between lumbar segments was collected at the peak of the disease. Spinal cords were individually homogenized in buffer containing PBS (0.01 M, pH 7.4), Tween 100 (0.1%), cocktail protease inhibitor, and phosphatase (*1:300, Sigma-Aldrich, EUA*). The homogenate was centrifuged at 10,000*g* for 5 min at 4°C. An aliquot of the supernatant was separated for the determination of proteins quantification by the Coomassie colorimetric method (*Bradford Reagent – Thermo Scientific, Rockford, IL, USA*) (51). The samples were normalized (1.2 µg/µL) and the concentration of the cytokines was determined by MULTIPLEX, xMap method, using the commercial kit (*#MCYTOMAG, Millipore, USA*). Reading was performed by the equipment Luminex 200 - Software xPonent/Analyst version 4.2.

### Cell Analyses by Flow Cytometry

The lymph nodes (pool of inguinal and mesenteric) were collected on the 7^th^ day after immunization. The cells were isolated by flushing the tissue through a cell strainer (70 μm) with RPMI medium, followed by centrifugation at 450 g for 5 min at 4 °C and the pellet was resuspended in 3 ml of RPMI medium. In a 24-well plate, the cells were cultured at a concentration of 1 × 10^6^ cells in 500 μl in RPMI medium with or without MOG_35–55_ (1 µg/ml) and BD GolgiStop™ Protein Transport Inhibitor (provided in the kit or Cat #554724) for 12 h. After 8 h, cells were stimulated with PMA (50 ng/ml) and ionomycin (1 μg/ml) for 4 h. The cells were stained for surface markers and intracellular cytokine with Mouse Th17/Treg Phenotyping kit *(#560767, BD Biosciences*) according to the manufacturer’s directions. Samples were analyzed using a FACS Canto II Flow Cytometer (*BD Biosciences, San Diego, USA*) after the acquisition. Data analysis was performed using FlowJo VX.0.7r2 (*Tree Star, Ashland, OR*). The strategy for analysis and representative dot plots of the experiments are shown in the **Supplementary Material**.

### Protein Analysis by Western Blotting

At the peak and the 26^th^ day after EAE, the spinal cord was dissected, the tissue from a region that included lumbar segments was collected and individually homogenized in buffer containing Hepes-NaOH (1 M, pH 7.9), NaCI (1,54 M), EGTA (200 mM), Triton-X 100 (1 %), cocktail protease inhibitor and phosphatase (1:300, Sigma-Aldrich, EUA). The homogenate was centrifuged at 15,000*g* for 10 min at 4°C. An aliquot of the supernatant was separated for proteins quantification by Coomassie colorimetric method (Bradford-Thermo Scientific) (51).

Protein samples were separated (30 μg) by electrophoresis on polyacrylamide gel SDS-PAGE (10% or 12%) and transferred to nitrocellulose membranes. Incubation consisted of 1 h at room temperature with blocking solution containing 5% blocking agent (GE Healthcare) dissolved in Tris-Buffered Saline - 10% Tween 20 (TBS-T) followed by incubation with primary antibodies Anti-GFAP (1:5,000, #Ab53554, *Abcam*), anti-CD11b (1:1,000, #Ab9485, *Abcam*), anti-MBP (1:500, #ab40390) or anti-GAPDH (1:5,000, #Ab75476, *Abcam*) overnight at 4°C in TBS-T/5% bovine serum albumin (BSA). Then, membranes were washed 3 times for 5 min in TBS-T and incubated for 1 h with secondary antibodies conjugated with peroxidase (GE Healthcare Life Science) at 1:5,000 in 5% blocking agent (GE Healthcare Life Science) dissolved in TBS-T. Bands were visualized using ECL (Pierce) solution with a digital image capture system and an optical density was determined by image lab software (UVITEC Cambridge).

### Histological Evaluation

At the peak of EAE clinical signs, the spinal cord was collected and fixed in 10% (w/v) paraformaldehyde (PFA) for at least 24 h, at room temperature. Following fixation, the tissues were transferred to 70% ethyl alcohol, dehydrated, embedded in paraffin, and sectioned (5 μm) using a microtome. The sections were stained with hematoxylin and eosin (HE) according to the standard protocol. The microscopic images were obtained using a DMLB microscope (Leica Microsystems) coupled to a camera (DFC420; Leica Microsystems, Switzerland) with software LAS version 4.5 (Leica Microsystems). The histological images from the spinal cord of each animal were blindly scored from 0 to 4 (inflammatory score), as follows: 0, no infiltration of inflammatory cells; 1, mild cellular infiltration; 2, moderate infiltration; 3, severe infiltration; and 4, massive infiltration (52, 53).

### Data Analysis

Statistical analysis of behavior test for mechanical hypernociception was carried out using a two-way analysis of variance (ANOVA) followed by Tukey’s post-test, whereas one-way ANOVA followed by Tukey’s t-test applied for AUC and biomolecular assays. All data are expressed as mean ± SEM. Statistical significance was set at *p* < 0.05. These analyzes were performed at Prism software, version 6.0 (GraphPad Software, La Jolla, CA).

## Results

### A Single Administration of CTX:SBA-15 Improves the Clinical Condition Induced by the EAE Model

**Figure 1:**
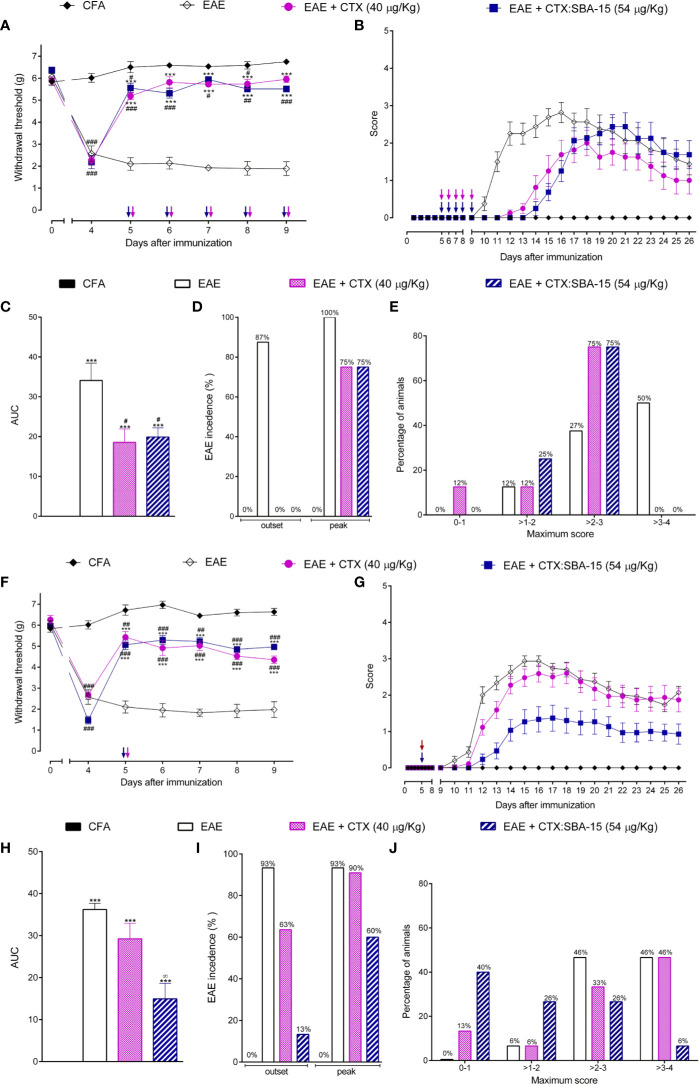
Effect of CTX:SBA-15 treatment on EAE in rodents. Nociceptive threshold was daily assessed throughout the experimental protocol (day 0) **(A, F)**. Animals were daily evaluated for the onset of clinical signs according to the scale parameters graded from 0 to 5 **(B, G)**. Statistical analysis of clinical signs was made by area under the curve analysis **(C, H)**, the incidence of the disease was shown in percentage (%) of animals showing clinical score equal to or greater than 1 in the group **(D, I)** and the maximum score reached for each animal on the evaluated period was shown in percentage (%) **(E, J)**. Results are expressed as mean (± SEM) n = 8 animal per group. Data are representative of two independent experiments. ***p < 0.001 indicates significant difference when compared to the EAE group. ^#^p < 0.05, ^##^p < 0.01 and ^###^p < 0.01 indicates a statistically significant difference when compared to CFA group. ∞p < 0.01 indicates a statistically significant difference when compared with EAE + CTX group. Two-way ANOVA test was used for behavior analysis, and a One-way ANOVA test was used for AUC, both followed by Tukey’s test.

We previously described that the repetitive administration of CTX (40 μg/kg), starting from the 5^th^ day post-immunization (5 consecutive doses 40 μg/kg) was effective in reducing EAE-induced clinical signs (45). Since SBA-15 conjugation enables an increase of 35% in CTX non-toxic dosage, a dose of 54 μg/kg was used in the present study (37). CTX:SBA-15 induced analgesic effect during the observed period when compared to the EAE group (**Figure 1A**). No differences were detected between unconjugated CTX and CTX:SBA-15 groups. Regarding the clinical scores, each animal was evaluated from the 9^th^ to 26^th^ day after immunization, (**Figures 1B–E**). The area under the curve (AUC) of clinical signs (individually calculated but represented as the mean of the group) (**Figure 1C**), the incidence of clinical signs (**Figure 1D**), and the maximum score reached by each animal (**Figure 1E**) were analyzed. The animals with EAE had the onset of clinical signs on the 9^th^ day after immunization (**Figure 1B**) and reached the peak between the 14^th^ to 18^th^ day after immunization. Unconjugated CTX and CTX:SBA-15 delayed the appearance of clinical signs (12th to 14th day after immunization), which had a milder clinical picture during the whole period when compared to the EAE + PBS group (**Figures 1B, C**). In addition, the disease incidence, as well as the intensity of the clinical signs were 25% lower in both treated groups (**Figures 1D, E**).

Since silica SBA-15 potentiates the immune response and prolongs the effect of various compounds, mice were treated with a single administration of unconjugated CTX or CTX:SBA-15 on the 5^th^ day after immunization. Interestingly, CTX in both conditions induced a long-lasting analgesic effect (**Figure 1F**), however, only CTX:SBA-15 delayed the manifestation of clinical signs from the 10^th^ to the 14^th^ day and improved the clinical scores (**Figures 1G, H**), also decreasing the intensity of motor impairments (**Figure 1J**). Among the animals treated with the complex CTX:SBA-15, only 13% of the animals at the outset and 60% at the peak of the disease had manifested the disease, showing around 30% less incidence of EAE (**Figure 1I**). Given these results, only a single administration of unconjugated CTX or CTX:SBA-15 was used for the next experiments.

### CTX:SBA-15 in a Single Administration Prevents the Muscle Impairment Induced by the EAE Model

**Figure 2:**
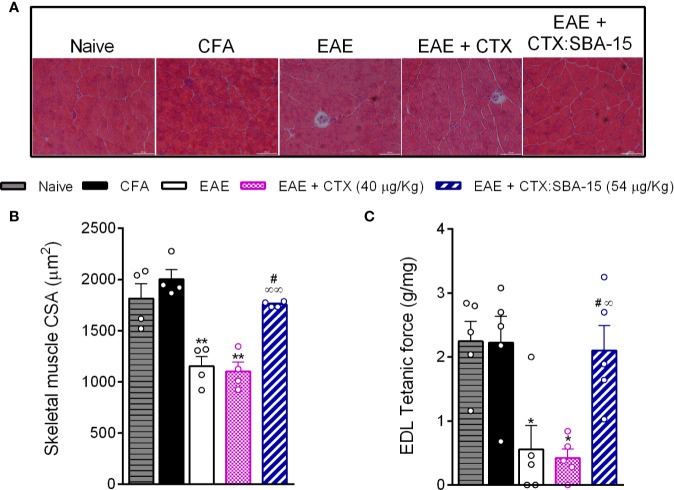
Effect of CTX: SBA-15 treatment on EAE-induced changes in muscle morphology and function. On the peak of the disease, the tibialis anterior muscle was collected for morphological analysis of the cross-sectional area **(A, B)** and the long digital extensor muscle (LDE) for muscle function **(C)**. Results are expressed as mean (± SEM) n = 4–5 animal per group. *p < 0.05 and **p < 0.01 indicates a statistically significant difference when compared to the naive and CFA groups. ^#^ *p* < 0.05 indicates a statistically significant difference when compared to EAE group. ∞*p* < 0.05 and ∞∞*p* < 0.01 indicate statistically significant differences when compared to EAE + CTX group. One-way ANOVA test was used, followed by Tukey’s test.

Since the EAE group treated with CTX:SBA-15 showed a decrease in the intensity of motor impairments, functional and morphological properties of skeletal muscle were also investigated at the peak of the disease (**Figure 2**). EAE animals displayed reduced body mass when compared to control groups (Naive and CFA). These changes were accompanied by decreases in the Soleus, Plantaris, Gastrocnemius and tibialis anterior muscle mass in animals (**Supplementary Figure 1**). Of interest, a single administration of CTX:SBA-15, but not unconjugated CTX, was sufficient to prevent the weight and skeletal muscle loss seen in EAE animals (**Supplementary Figure 1**). In addition, the tibialis anterior muscle atrophy was apparent at the single fiber level, measured by myofiber cross-sectional area. The treatment with CTX:SBA-15, but not unconjugated CTX, showed a protective effect against EAE-induced skeletal muscle atrophy, preserving myofibers cross-sectional area of tibialis anterior muscle as compared to EAE group (**Figures 2A, B**).

To evaluate the skeletal muscle contractile property, we assessed the maximal tetanic absolute force of the extensor digitorum longus muscle (EDL). As expected, the EAE showed a reduction of this property, when compared to control groups. The treatment with CTX:SBA-15, but not unconjugated CTX, showed a protective effect against EAE-induced skeletal muscle functional loss (**Figure 2C**). Thus, these findings reinforce that the treatment with a single administration of CTX:SBA-15 prevents EAE-induced motor impairment.

### CTX:SBA-15 Decreases Peripheral and Central IL-17 Expression

**Figure 3:**
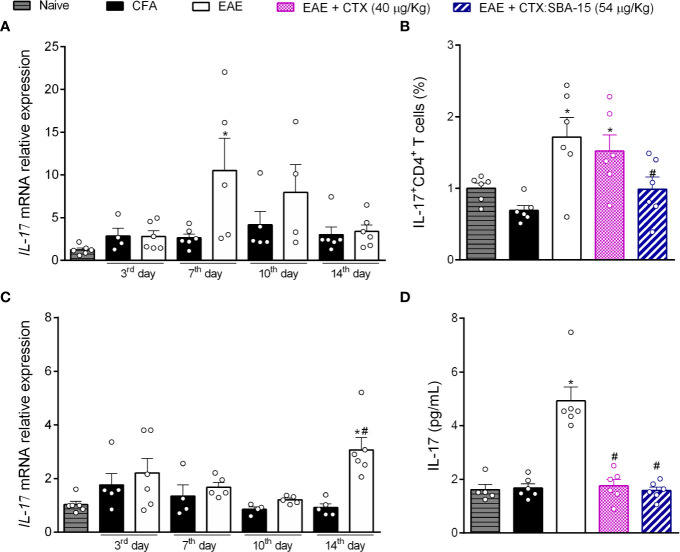
The treatment with CTX:SBA-15 decreases the expression of Th17 cells and IL-17 cytokine induced by EAE. The lymph node **(A)** and spinal cord **(C)** samples were collected at indicated times for mRNA for IL-17. At 7^th^ day after immunization, the cells from lymph nodes were extracted and stained for extracellular marker, for CD4^+^, and intracellular cytokine IL-17 **(B)**. The results were evaluated by flow cytometry and analyzed in FlowJo software. At the peak of disease the samples from the spinal cord were processed to the protein assay for IL-17 **(D)** assessed by MULTIPLEX. All data are expressed as mean (± SEM) n = 5–6 animal per group. *p < 0.05 indicates a statistically significant difference when compared to the Naive and CFA group. ^#^p < 0.05 indicates a statistically significant difference when compared to the EAE. One-way ANOVA test was used, followed by Tukey’s test.

Considering that a single administration of the CTX:SBA-15 complex improves the clinical signs and muscle functions, we investigated its effect in peripheral lymphoid organs (lymph node) and at the CNS (spinal cord), regions that are deeply involved in the disease development. Firstly, the time course of IL-10 (**Supplementary Figure 2A**) and IL-17 mRNA expression in lymph nodes (**Figure 3A**) were evaluated in EAE animals. The results showed that EAE induced a significant increase in pro-inflammatory cytokine IL-17 mRNA expression in the lymph node, on the 7^th^ day after immunization (**Figure 3A**); no differences were detected in other time points or IL-10 mRNA expression (**Supplementary Figure 2A**). Considering these results, the effect of the treatments on the expression of Th17 cells at the same time point was evaluated, through the CD4^+^/Foxp3^+^(**Supplementary Figure 2B**) or CD4^+^/IL-17^+^ (**Figure 3B**) expression by flow cytometry (gating strategy analysis and representative Dot Plots, **Supplementary Figure 3**). The results showed an increase of Th17 cells in the EAE and EAE + CTX groups, while the EAE animals treated with CTX:SBA-15 showed a significant reduction of Th17 cells (**Figure 3B**). No difference was observed in Treg cell analysis (**Supplementary Figure 2B**).

Taking into account the effect of CTX:SBA-15 on the lymph node, and the relevance of interleukin IL-17 as a key player in the development of the disease, we investigated if this interference would also occur at the CNS (spinal cord). Our data shows that EAE induces an increase in the IL-17 mRNA expression in the spinal cord, at the peak of the disease (**Figure 3C**). In addition, it was observed that EAE mice showed an increase in the IL-17 interleukin release at this same period by multiplex, while a single administration of CTX or CTX:SBA-15 significantly reduced this interleukin to normal levels (**Figure 3D**). Both mRNA expression and multiplex analyses were also performed for interleukin IL-10 (mRNA and cytokine level). An increase of IL-10 mRNA expression in the spinal cord was only observed at the peak of the disease in EAE animals, but there was no difference in the interleukin levels (**Supplementary Figures 2C, D**).

### CTX:SBA-15 Single Administration Reduces the Neuroinflammation in the Spinal Cord

**Figure 4:**
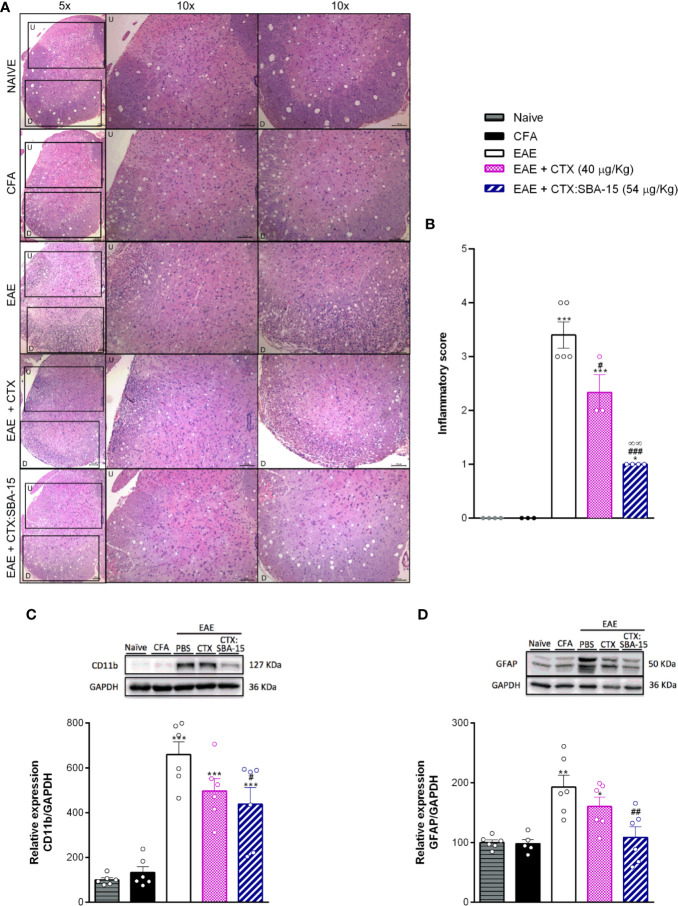
Effect of the treatment with CTX:SBA-15 on neuroinflammation. Animals were treated with CTX (40 µg/kg, s.c.) or CTX:SBA-15 (54 µg/kg, s.c.) in a single administration on the 5^th^ day after immunization with MOG_35-55_ in CFA. The spinal cord of the animals was collected at the peak **(A–C)** or at 26° after immunization **(D)**. The cell infiltration analysis was performed by hematoxylin and eosin (HE) staining. Representative sections of cell infiltration are shown **(A)** as well histological scores **(B)** of whole spinal cord. Upper (U) and down (D) indicate the magnified corresponding figures. Scale bar 100 μm (n = 4-5) animals per group). The expression of microglia, CD11b **(C)**, or astrocytes, GFAP (D), markers, were analyzed by Western Blotting assay (n = 6 animals per group). All data are expressed as the mean (± SEM). ***p<0.001, **p<0.01 and *p<0.05 indicates a statistically significant difference when compared to the Naive and CFA group. ^#^p<0.05, ^##^p<0.01 and ^###^ *p*<0.001 indicates a statistically significant difference when compared to the EAE. ∞∞*p*<0.01 indicates a statistically significant difference when compared to EAE + CTX group. One-way ANOVA test was used, followed by Tukey’s test.

Considering that the EAE model induces a massive immune cell infiltration to the CNS, together with glial cells activation/proliferation, it was next evaluated whether CTX:SBA-15 treatment could also attenuate those activities in the spinal cord. Cell infiltration was evaluated using histological scores in spinal cord sections stained with HE. The results indicate that the number of infiltrating cells was increased in spinal cord sections of the EAE group, mainly observed in the dorso-lateral and ventral areas of spinal cord, when compared to control groups (CFA and naïve), while unconjugated CTX and CTX:SBA-15 decreased EAE-induced cellular infiltration (**Figures 4A, B**). Of interest, CTX:SBA-15 treated mice displayed a more pronounced drop in cell numbers when compared to unconjugated CTX (**Figure 4B**).

Through western blotting assays, at the peak of the disease, a significant increase of microglial/macrophage marker (CD11b) was observed in the EAE animals when compared to controls groups (CFA and naïve) (**Figure 4C**), while no differences were observed for the analysis of GFAP expression at this period (data not shown). Although there is no difference in the relative expression of CD11b marker when comparing unconjugated CTX and CTX:SBA-15 groups, CTX:SBA-15 in a single administration, but not unconjugated CTX, partially reduces the EAE-induced CD11b expression (**Figure 4C**). At the 26^th^ day after immunization a significant increase of astrocyte marker (GFAP) was observed in EAE-PBS-treated animals (**Figure 4D**); in contrast, no difference was observed in CD11b expression (data not shown). Again, unconjugated CTX did not interfere with this expression, while the animals treated with CTX:SBA-15 show reduced GFAP expression (**Figure 4D**) compared to the EAE group, despite of the fact that there is no difference in this expression between unconjugated CTX and CTX:SBA-15 groups.

## Discussion

MS is a chronic inflammatory autoimmune disease that especially affects the CNS. Demyelination lesions and neuroinflammation mostly on the brain and spinal cord are the pathological hallmarks of this disease (2, 54, 55). Considering the large variability of clinical manifestation and disease progression, the treatment is complex, based on drugs that slow the progression and manage the symptoms. Unfortunately, there is no cure for this condition yet (17, 18, 56).

To better understand the pathology and the sensorial and motor alterations, as well as to find new therapeutic compounds, previous data from our group confirmed the antinociceptive and immunomodulatory effects of CTX in the MS model (EAE) induced in mice. These results showed that CTX, when administered in 5 consecutive doses, controlled both pain sensitivity and clinical signs development, while when administered in a single administration, it interfered with the pain sensitivity without any effect on clinical signs (45). However, CTX is a neurotoxin, and its use, especially in high doses or in chronic schedules, is limited by its toxic effect, mainly for debilitating conditions. Hence, taking advantage of the silica SBA-15 beneficial effects as a carrier agent (37, 57), we previously demonstrated that SBA-15 attenuates CTX-induced toxic effects by increasing in 35% the CTX lethal dose 50% (LD_50_) (37). Thus, this work aimed to improve the immunomodulatory effect of CTX through its adsorbing to SBA-15 in the EAE model. Both treatments with unconjugated CTX or CTX:SBA-15, administered in 1 or 5 consecutive doses, induced analgesic effect in the EAE model during the evaluated period. Corroborating previous results about CTX, regarding the motor alterations (clinical sings), 5 consecutive administrations of unconjugated CTX delayed the manifestation of clinical signs and lowered the intensity of motor impairment (45) which was also observed with CTX:SBA-15 treatment. In contrast, when administered in a single administration, while unconjugated CTX does not affect the motor symptoms, the CTX:SBA-15 complex delayed the onset of symptoms and attenuated the intensity of clinical signs. A recent clinical report suggests that starting the treatment during the early symptoms with a highly efficient drug is the best option to prevent the progression of the irreversible lesions and change the disease course (58). However, this has never been seen with a single administration treatment for MS patients or even in animal models of MS.

Although MS has been considered as mainly a neuroinflammatory disease with demyelination and additional axonal damage in the CNS, lower motor activity and slow conduction in peripheral sensory and motor nerve fibers have also been observed (59–61). Reports have shown that EAE induces atrophy in addition to disruption in morphology (14, 62) and disfunction in skeletal muscles (63). Our results demonstrate that mice with EAE showed a reduction in body weight, in part as a consequence of the muscular mass loss and atrophy. Surprisingly, the treatment with CTX:SBA-15, but not with unconjugated CTX alone, prevents EAE-induced muscle atrophy and functional loss, maintaining the health contractile properties of the EDL muscle. It was described that the atrophy observed in the EAE model is related to sudden muscle inactivity due to neurological injuries, which may be linked to T cell infiltration and microglial activation (14, 60, 63). The present results confirm the effectiveness of a single administration of CTX:SBA-15 in the prevention of EAE-induced motor impairment, probably as a result of the decrease in the immune response and neuroinflammation.

Classical MS immune responses are initiated with antigen-bearing dendritic cells, which migrate to lymphoid organs, such as the lymph nodes, leading to activation and polarization of naive CD4^+^ T cells (64, 65). Increasingly clinical and pre-clinical studies demonstrate the importance of Th17 cells in the MS pathogenesis. High levels of IL17 in the serum and cerebrospinal fluids were found in patients with MS, which may be related to the disease progression and the extension of the lesions measured by magnetic resonance imaging. Th17 contributes to the neuroinflammation by recruiting immune cells and increasing the release of the pro-inflammatory cytokines, in addition to inducting BBB breach (66–69). Our results demonstrating that single administration of CTX:SBA-15 reduces IL-17 levels and inhibits the Th17 cell recruitment point out that CTX:SBA-15 complex may exert its long-lasting effects through IL-17 inhibition. It was previously demonstrated that the inhibition of Th17 cells in lymph organs can reduce the severity of EAE, in addition to the fact that knockout mice (*Il17a^−/−^* mice) are partially resistant to EAE induction (70).

The neuroinflammation induced by MS, in particular by Th17 cells, leads to an increase in BBB permeability, which allows the influx of inflammatory cells into the CNS, activating resident glial cells, such as microglia and astrocytes. Also, astrocytes may contribute to immunomodulation and protection of neurons against cytotoxins and oxidants, and microglia/macrophages activation plays a fundamental role in neuroinflammation. These events mediate the recurrent episodes of demyelination and axonal lesion (4, 67, 71). Some evidence suggests that there is integration between T cells and microglia, as well as astrocytes, which have receptors for IL-17, and the inhibition of those cells can reduce the intensity of EAE (72–75). Our results, showing that CTX:SBA-15 decreased the infiltrating cell in the CNS and the central expression of glial cells induced by EAE, reinforce the ability of SBA-15 in enhancing the immunomodulatory effects of CTX. Besides that, CTX:SBA-15 prevents the EAE-induced decrease in the myelin basic protein (– MPB) expression, observed in a later period in the EAE groups treated with PBS or even with unconjugated CTX (**Supplementary Figure 4**), suggesting that CTX:SBA-15-induced downregulation in neuroinflammation and immune response would interfere in the demyelination process.

The presented data corroborate the mechanism of unconjugated CTX, as previously described by our group (45). On the other hand, the increase in CTX dose together with silica properties promoted even higher benefits in the EAE model with a single administration in the early phase of development. In addition, this work evidenced the CTX:SBA-15 control over peripheral important functional and morphological parameters of skeletal muscle altered by the disease, as well as its effect on central inflammation.

The properties of nanostructured silica in to carry, to protect, and to deliver the entrapped antigens or compounds are attributed to its hexagonal nanostructured pores, which protect compounds against proteolysis, organic solvents, high shear stresses, and low pH (57, 76, 77). Previous data from our group demonstrated that silica does not alter some of the mediators known to be responsible for the antinociceptive effect of CTX (37). Considering that, the fact that once encapsulated and kept entrapped in the silica, the compound may have its release controlled (34, 78) could be a possible explanation for the SBA-induced CTX effect potentiation in EAE animals. However, the lack of pharmacokinetic studies is a limitation of the present study, and future assays should be performed to address this question. Importantly, silica is an inert particle and, based on the present results, would have its use possibly expanded to other therapeutic compounds in order to decrease the required treatment schedule, primarily for medicines with undesirable effects.

In summary, one single administration of the complex CTX:SBA-15 in the early phase of development can ameliorate the EAE-induced neuroinflammation, through the prevention of peripheral Th17 cells recruitment, reduction of cells infiltrate to the CNS, decrease in spinal cytokine IL-17 expression and glial cells activation, hence reducing the intensity and incidence of clinical signs, and preserving muscle mass and function. Thus, on the whole, our results showed that SBA-15 when used as a carrier, besides enabling the increase in CTX dose, can improve the immunomodulatory and anti-inflammatory effects of this toxin, suggesting that the complex CTX:SBA-15 could be a potential formulation for the control of sensitivity, such as pain, motor alterations, atrophy and loss of motor function. Moreover, these findings suggest that SBA-15 can be a useful tool for new therapeutic formulation for several biological compounds.

## Data Availability Statement

The raw data supporting the conclusions of this article will be made available by the authors, without undue reservation.

## Ethics Statement

The animal study was reviewed and approved by Institutional Animal Care Committee of the Butantan Institute (CEUAIB, protocol number 1320/14 and 7986010819).

## Author Contributions

MS’A, GP, and OS’A designed the study. MS’A, AG, and FL performed the experiments and analyzed the data for study design. NT helped with EAE induction. MS’A and MR performed and analyzed the skeletal muscle experiments. MS’A and AB performed and analyzed the PCR assays. MB, LK and WC helped with the collection, preparation, and strain of cells for flow cytometry assay. OR helped with analysis and interpretation of Flow Cytometry data. JF and VZ helped with the design study and results interpretation. MS’A and GP wrote the original draft with input from OS’A, LK, JF, and VZ. GP and OS'A obtained the funding for the study. All authors contributed to the article and approved the submitted version.

## Funding

This work was supported by funds from São Paulo Research Foundation (FAPESP), grants #2014/19397-0, #2015/01254-1, #2017/17844-8 and #2013/07467-1 (CETICs program), and this study was financed in part by the Coordenação de Aperfeiçoamento de Pessoal de Nível Superior-Brazil (CAPES) - Finance Code 001.

## Conflict of Interest

The authors declare that the research was conducted in the absence of any commercial or financial relationships that could be construed as a potential conflict of interest.
