# Supplementary material for: The Crotoxin:SBA-15 Complex Down-Regulates the Incidence and Intensity of Experimental Autoimmune Encephalomyelitis Through Peripheral and Central Actions
Source: Front Immunol. 2020 Oct 28;11:591563. doi: 10.3389/fimmu.2020.591563 (PMC7655790; doi:10.3389/fimmu.2020.591563)
Supplement: Supplementary file 1 [file DataSheet_1.pdf]

Supplementary Material

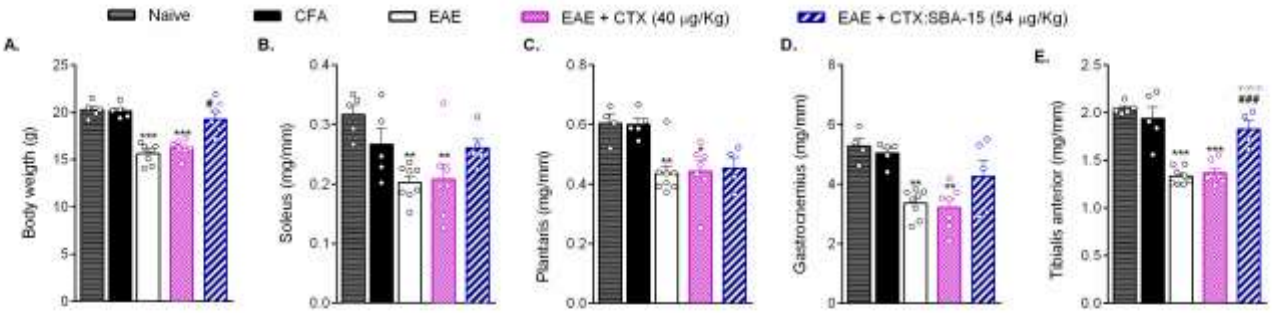

Supplementary Figure 1. Evaluation of the body and muscle mass in EAE mice treated with CTX: SBA-15. On the peak of the disease, the animals were weighed **(A)** and the skeletal muscles of the hind limb were collected for mass analysis **(B-E)**. Results are expressed as the mean (± SEM) n = 4-8 animals per group. * p<0.05, ** p<0.01 and *** p<0.001 indicates a significant difference when compared to the naive and CFA groups. # p<0.05 and ### p<0.05 indicates significant difference when compared to EAE group. ∞ p<0.05 indicates significant difference when compared to EAE + CTX group. One-way ANOVA test was used, followed by Tukey's test.

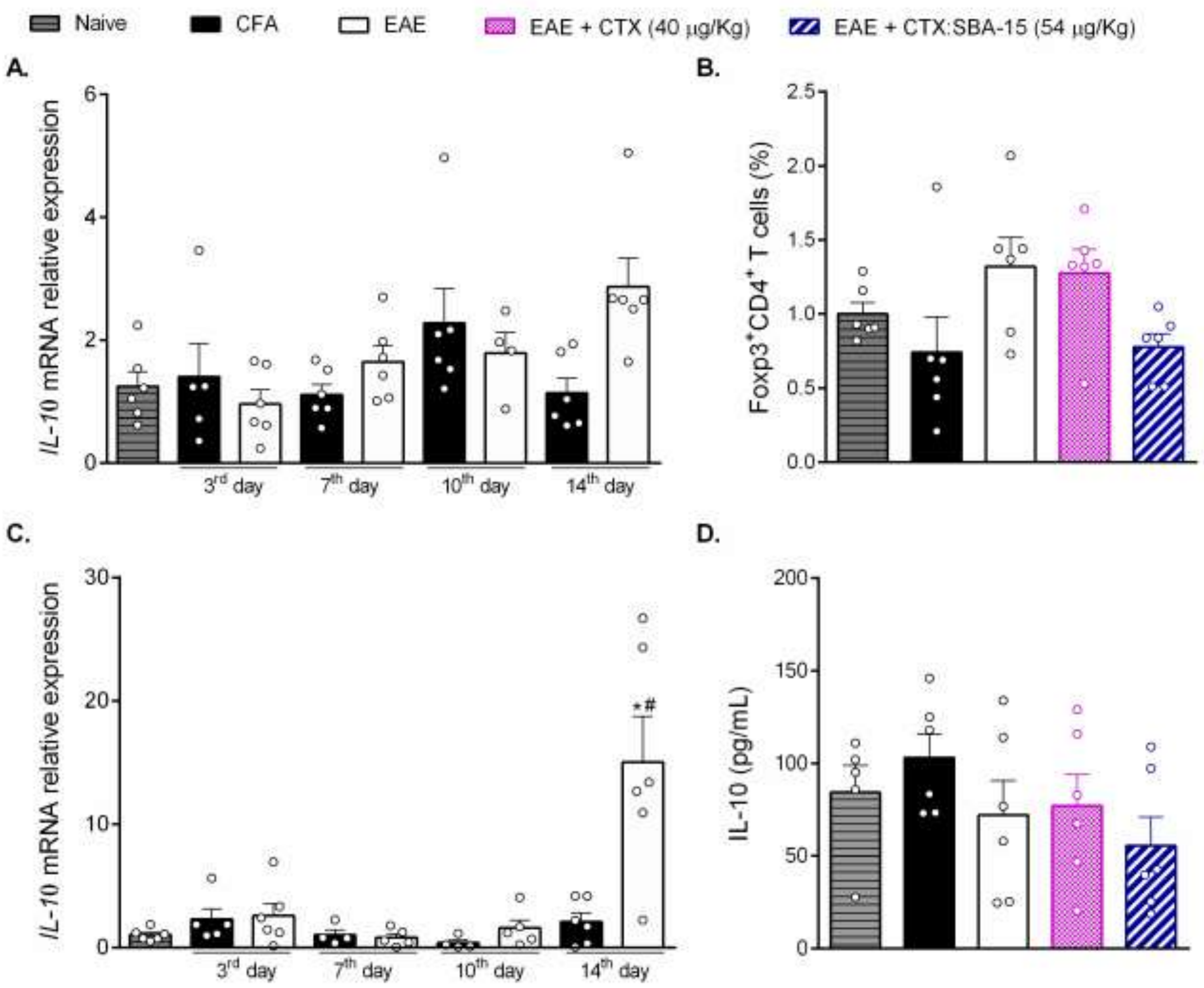

Supplementary Figure 2. Evaluation of the treatment with CTX or CTX: SBA-15 on Treg cells and IL-10 cytokine in animals submitted to EAE. The animals were immunized and treated with 1 dose of CTX (40 µg/kg, s.c.) or CTX: SBA-15 (54 µg/kg, s.c.) on the 5th day after EAE induction. The lymph nodes and spinal cord was collected on 3rd, 7th, 10th and 14th (peak) day for mRNA expression assay. The mRNA of the cytokine IL-10 in lymph nodes **(A)** and spinal cord **(C)** was evaluated by RT-PCR. At 7th day after immunization the cells from lymph nodes were extracted and stained for extracellular marker, for $CD4^+$, and intracellular Foxp3 **(B).** The results were evaluated by flow cytometry and analyzed in FlowJo software. Assessment of IL-10 cytokine expression **(D)** was performed by MULTIPLEX assay. Results are expressed as the mean (± SEM) n = 5-6 animals per group. * $p<0.01$ indicates significant difference when compared with the naive and CFA groups. $^{\#}$ $p<0.05$ indicates significant difference when compared to EAE group. One-way ANOVA test was used, after Tukey's test.

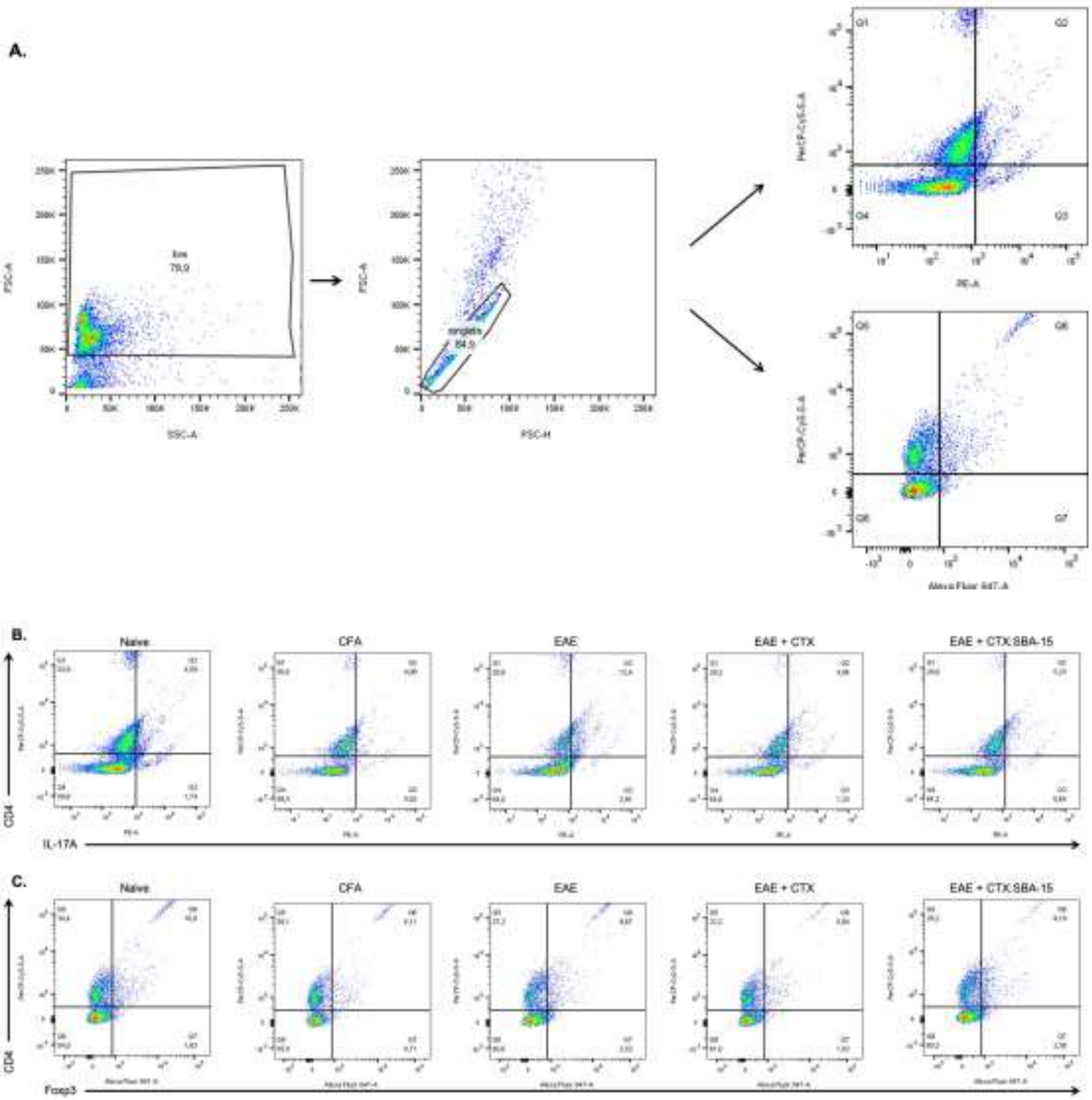

Supplementary Figure 3. Schematic flow cytometry gating strategy analysis **(A)**. Dot Plots, representative image of the animals with EAE and EAE + CTX:SBA-15, and TCD4$^+$ cell frequency producing the respective cytokines, IL-17 **(B)** and Foxp3 **(C)**, expressed by the results (fig 3B and suppl fig 2B).

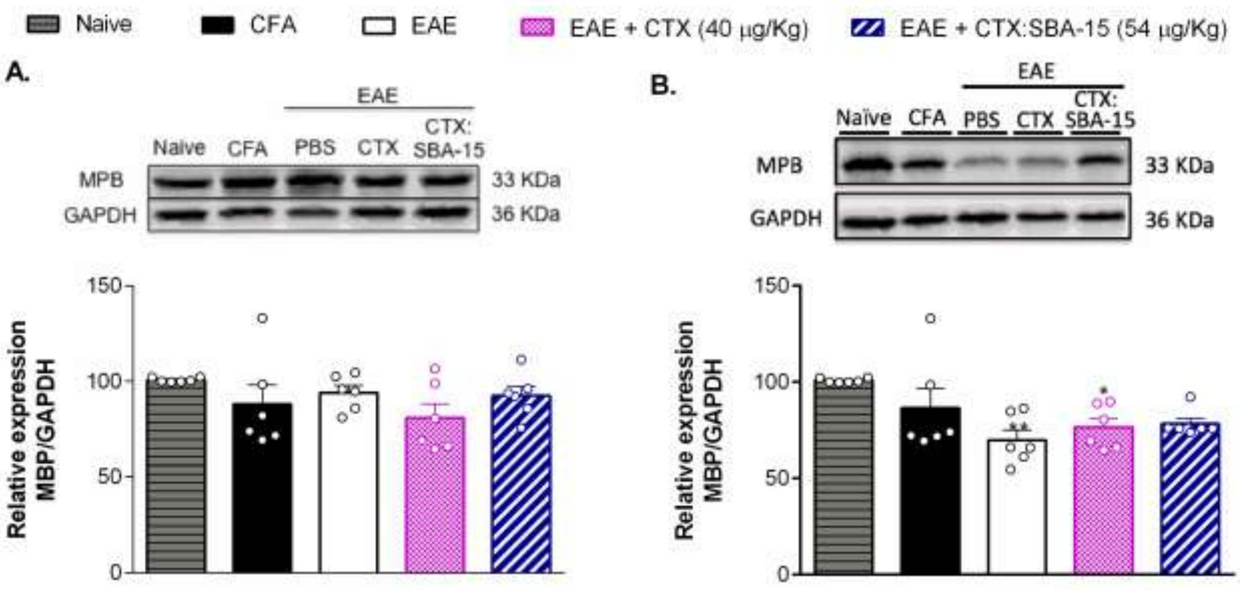

Supplementary Figure 4. Effect of unconjugated CTX or CTX:SBA-15 on MBP expression in EAE animals. Assessment of MPB expression was performed by Western Blotting assay. Animals were immunized and treated with 1 dose of CTX (40 µg/kg, s.c.) or CTX: SBA-15 (54 µg/kg, s.c.) on the 5th day after EAE induction. The spinal cord was collected on 26th **(A)** or 40th day **(B)** after immunization. Results were normalized using GAPDH. Results are expressed as the mean (± SEM) n = 6 animals per group. ** $p<0.01$ and * $p<0.05$ indicates a significant difference when compared to the Naive group. One-way ANOVA test was used, after Tukey's test.
